# “Tekenscanner”: a novel smartphone application for companion animal owners and veterinarians to engage in tick and tick-borne pathogen surveillance in the Netherlands

**DOI:** 10.1186/s13071-019-3373-3

**Published:** 2019-03-26

**Authors:** Frans Jongejan, Suzanne de Jong, Timo Voskuilen, Louise van den Heuvel, Rick Bouman, Henk Heesen, Carlijn Ijzermans, Laura Berger

**Affiliations:** 1 Utrecht Centre for Tick-borne Diseases (UCTD), FAO Reference Centre for Ticks and Tick-borne Diseases, Faculty of Veterinary Medicine, Utrecht University, Yalelaan 1, 3584 CL Utrecht, The Netherlands; 2 Vectors and Vector-borne Diseases Research Programme, Department of Veterinary Tropical Diseases, Faculty of Veterinary Science, University of Pretoria, Private Bag X04, Onderstepoort, 0110 Republic of South Africa; 3 Bayer Animal Health, Energieweg 1, 3641 RT Mijdrecht, The Netherlands

**Keywords:** Smartphone app, Companion animals, Ticks, Tick-borne pathogens, The Netherlands

## Abstract

**Background:**

The engagement of companion animal owners into the process of collecting epidemiological data can be facilitated through smartphone applications. In April 2018, the “tekenscanner“ (Dutch for tick scanner) app was launched with the aim of engaging pet owners and veterinarians to record ticks removed from their pets and submit these ticks for identification and pathogen testing. Tick-borne pathogens identified in ticks removed from dogs and cats during the first 6 months after the app was launched in the Netherlands are reported.

**Methods:**

The tekenscanner app was used to record the geographical coordinates of ticks removed from dogs or cats onto a map of the Netherlands. A barcode was assigned to each tick for the easy tracking of each submission to our laboratory for taxonomic identification. Thereafter, DNA extracted from the ticks was PCR amplified, subjected to reverse line blot hybridization (RLB) and screened for a broad range of tick-borne pathogens. Results were added to the same app, usually within 2 weeks after the submission of each tick.

**Results:**

The app was downloaded 5591 times and resulted in the collection of 1273 georeferenced and barcoded ticks, with a peak submission in May and June of 2018. There were 1005 ticks collected from 406 dogs and 268 ticks collected from 111 cats. *Ixodes ricinus* was the predominant species (90.0%), with all stages found on dogs as well as on cats. *Ixodes hexagonus* (7.3%) female and nymphal ticks were also identified on both hosts, whereas adults of *Dermacentor reticulatus* (2.4%) and *Rhipicephalus sanguineus* (0.2%) were exclusively found on dogs. Nearly 15% of the ticks recovered from dogs carried one or more pathogens, whereas 13.8% of the ticks removed from cats were infected. *Ixodes ricinus* collected from dogs contained *Borrelia* spp. (1.9%), *Babesia* spp. (0.7%), *Anaplasma phagocytophilum* (1.3%), “*Candidatus* Neoehrlichia mikurensis” (2.9%) and *Rickettsia helvetica* (7.3%). *Ixodes ricinus* recovered from cats were infected with *Borrelia* spp. (1.9%), *Babesia* spp. (0.4%), *A. phagocytophilum* (1.9%), “*Ca.* Neoehrlichia mikurensis” (2.6%) and *R. helvetica* (6.7%). *Ixodes hexagonus* ticks (*n* = 93) were not infected. *Dermacentor reticulatus* ticks, found only in autumn, were infected with *Rickettsia raoultii* (16 %) and *A. phagocytophilum*. Three *R. sanguineus*, on dogs from France and the USA imported into the Netherlands, were all negative.

**Conclusions:**

The tekenscanner app is a versatile tool to use for submission of ticks and facilitated the fast feedback of test results. Community engagement through the app is suitable for identifying hotspots for ticks and tick-borne pathogens and provided an early warning system for exotic ticks invading the Netherlands.

## Background

Ticks (Acari: Ixodidae) are important vectors of a broad range of pathogens affecting both human and animal health worldwide [1]. For companion animals, the importance of ticks as vectors of *Babesia*, *Borrelia*, *Anaplasma* and *Ehrlichia* species has been well documented [2]. Moreover, changes in the epidemiology due to climate and tick habitat modifications as well as increasing host availability and movement of people with their companion animals, increases the importance of tick surveillance [3]. This has resulted in studies targeting ticks on companion animals in many areas throughout western Europe. For instance, surveillance of ticks from domestic dogs and/or cats has been conducted in nation-wide studies in the UK [4, 5], Belgium [6], Germany [7], Poland [8], as well as in a multinational European study including Hungary, France and Italy, Germany and Belgium [9].

In the Netherlands, monitoring of tick-borne diseases in dogs started in 2004 when outbreaks of autochthonous *Babesia canis* infections affected 23 dogs, including four fatal cases [10]. The prediction that the introduction of *Dermacentor reticulatus* ticks into the Netherlands may result in the establishment of permanent resident populations has become a reality. This was recently confirmed with the identification of novel foci of *D. reticulatus* in the Netherlands as well as in Belgium [11]. The outbreak of babesiosis in dogs was a starting point for a national campaign promoted by the Royal Netherlands Society of Veterinary Medicine (KNMvD). Over 200 veterinary clinics throughout the Netherlands were requested to submit ticks collected from their companion animal patients to our research centre. Information packages including brochures and collection tubes were widely distributed to facilitate the submission of ticks. From the results obtained with the first 4298 ticks collected in 2005 and 2006, it was concluded that a much broader spectrum of ticks and tick-borne pathogens, including several zoonotic pathogens, was present in the Netherlands than previously thought [12]. Over the past 10 years, veterinarians have continued submitting ticks removed from their companion animal patients and all of these ticks were taxonomically identified as a free service, but they were only tested by PCR upon request.

It is interesting to compare our approach with similar initiatives conducted in other parts of Europe. For instance, in the UK it was realized that systematic surveillances of tick-borne diseases in companion animals are not routinely undertaken [3]. As a result, a large-scale surveillance programme was initiated whereby more than 1000 veterinary practices were recruited through a media campaign, resulting in 6555 tick samples from infested dogs over a period of 16 weeks in 2015 [4]. As part of the same campaign, 278 veterinary practices submitted ticks removed from cats, which eventually resulted in the identification of a range of *Babesia* and *Borrelia burgdorferi* (*sensu lato*) species [13]. Another pet-owner-based survey was conducted in Switzerland, where dog owners in a rural town were sent postal requests to send ticks from their dogs and cats over 2 consecutive years. In total, 3003 ticks were received for identification which had been removed by the owners from 249 dogs and 117 cats [14].

Both examples are in line with our experiences, which indicate that active involvement of the companion animal owner and/or veterinary community is essential to conduct surveys on ticks and tick-borne diseases on companion animals. Here, we take it one step further by creating a much closer link between the citizen science community and testing laboratories. To facilitate this, we introduced a novel smartphone app to engage companion animal owners as well as veterinarians into active surveillance activities targeting ticks and tick-borne diseases in the Netherlands. This app, named “Tekenscanner” (Dutch for “Tick scanner”) was launched in April 2018 and the results of the first 6 months are presented here.

## Methods

### Study design

After downloading the Tekenscanner app, users were asked to create an account and enter the age, sex and breed of their pet into their account. The geographical coordinates of the location where the tick was removed from the dog or cat was recorded and plotted onto a map of the Netherlands. For the next step, each participant received a sample submission set containing a tick tube and a barcoded letter with instructions how to send the sample to our laboratory (UCTD). After arrival, the barcode was scanned and used to track each submission through eLabjournal (Groningen, The Netherlands), an electronic laboratory notebook wherein all test procedures and results were recorded for each tick. Prior to DNA extraction, each tick was identified using a binocular microscope with 80× magnification while consulting a recent taxonomic reference book wherein all European ticks have been described in detail [15]. Through the tekenscanner app, pet owners could submit ticks and receive feedback about tick identification and infection status within a very short time interval (usually less than 2 weeks).

### DNA extraction

*Ixodes ricinus* ticks of the same stage (larvae/nymphs) and either males or females from the same host were pooled, with an average number of 4.5 ticks per pooled sample. All other ticks, such as *I. hexagonus*, *D. reticulatus* and *R. sanguineus* were tested individually. For DNA extraction, ticks were placed in sterile 2 ml microcentrifuge tubes containing 180 μl of lysis buffer and frozen at -20 °C. Thereafter, metal beads (5 mm in diameter) were added to the frozen samples, which were subsequently disrupted in a TissueLyser (Qiagen Benelux BV, Venlo, the Netherlands) at 50 Hz for 3 min. DNA was extracted from the triturated ticks using a GeneJet genomic DNA purification kit (Thermo Fisher Scientific, Landsmeer, the Netherlands) according to the manufacturer’s instructions. Extracted DNA was eluted in 150 μl of elution buffer, and either used directly or stored at -20 °C. After DNA extraction, DNA was PCR amplified and tested by reverse line blot hybridisation (RLB).

### PCR

For *Babesia/Theileria* species PCR, the primer pair RLB-F2 (5′-GAC ACA GGG AGG TAG TGA CAA G-3′) and RLB-R2 (5′-biotin-CTA AGA ATT TCA CCT CTG ACA GT-3′) was used to amplify the V4 variable region of the *18S* rRNA gene [16, 17]. The length of the PCR amplicon was 460 bp. For *Anaplasma/Ehrlichia* and *Rickettsia* PCR, the primer pair Ehr-F2 (5′-AGA GTT TGA TCC TGG CTC AG-3′) and Ehr-R2 (5′-biotin-GAG TTT GCC GGG ACT TYT TCT-3′) was used to amplify the V1 variable region of the *16S* rRNA gene [18]. The length of the PCR amplicon was 460–500 bp. For *Borrelia* PCR, the primer pair Bor-F (5′-ACC ATA GAC TCT TAT TAC TTT GAC CA-3′) and Bor-R (5′-biotin-GAG AGT AGG TTA TTG GCC AGG G-3′) was used to amplify the 5S-23S rDNA spacer region gene [19]. The length of the PCR amplicon was 180–230 bp. Each PCR was performed in a total volume of 20 μl, containing 10 μl of 2× Phusion Hot Start High Fidelity Master Mix (Thermo Fisher Scientific), 0.5 μM of each primer, 2 μl of extracted genomic DNA and the remaining volume was double-distilled water. PCR primers were purchased from Life Technologies Europe BV, Bleiswijk, the Netherlands.

As positive controls, genomic DNA from *B. canis*, *Babesia gibsoni*, *Ehrlichia canis*, *A. phagocytophilum* and *B. burgdorferi* was used. Distilled water was used as negative control.

### Reverse line blot (RLB) hybridization

Reverse Line Blot (RLB) hybridization assay has the advantage of being able to analyse multiple samples against multiple probes simultaneously, and it was first applied to differentiate tick-borne *Borrelia* species [19]. All probes used to differentiate *Babesia*, *Theileria* [20], *Anaplasma* and *Ehrlichia* [21] are listed in Table 1. Moreover, probes for the differentiation of *Rickettsia* species were also added to the membrane (Table 1) [22].

**Table 1:** Reverse line blot hybridization probe sequences with a C6 amino linker at the 5′ end

Probe	Sequence (5′–3′)	Reference
*Ehrlichia*/*Anaplasma* catch-all	GGGGGAAAGATTTATCGCTA	[21]
*Anaplasma phagocytophilum* (1)	GCTATRAAGAATARTTAGTGG	[36]
*Anaplasma phagocytophilum* (2)	TTGCTATRRAGAATARTTAGTGG	[37, 38]
*Anaplasma platys* (1)	CGGATTTTTGTCGTAGCTTGCTATGAT	[36]
*Anaplasma platys* (2)	GTCGTAGCTTGCTATGATA	[39]
*Ehrlichia canis* (1)	TATAGCECTCTGGCGGAAATTGGTTAG	[36]
*Ehrlichia canis* (2)	TCTGGCTATAGGAAATTGTTA	[37]
*Ehrlichia ewingii*	TTCCTAAATAGTCTCTGACTATTT	[40]
“*Ca.* Neoehrlichia mikurensis”	GCTGTAGTTTACTATGGGTA	[37]
*Theileria*/*Babesia* catch-all	TAATGGTTAATAGGARCRGTTG	[20]
*Babesia* catch-all (1)	ATTAGAGTGTTTCAAGCAGAC	[38]
*Babesia* catch-all (2)	ACTAGAGTGTTTCAAACAGGC	[38]
*Babesia caballi*	GTGTTTATCGCAGACTTTTGT	[20]
*Babesia canis* (1)	GGTTGGTTATTTCGTTTTCGC	This study
*Babesia canis* (2)	TGGTTGGTTATTTCGTTTTCG	[38]
*Babesia divergens*	ACTRATGTCGAGATTGCAC	[16]
*Babesia gibsoni* (1)	CTGCGTTGCCCGACTCG	[36]
*Babesia gibsoni* (2)	TACTTGCCTTGTCTGGTTT	[38]
*Babesia microti* (1)	GCTTYCGAGCGTTWTTTTATTG	This study
*Babesia microti* (2)	GRCTTGGCATCWTCTGGA	[38]
*Babesia rodhaini*	TGTGGATTAGTGCGCAAG	[38]
*Babesia rossi*	CGGTTTGTTGCCTTTGTG	[41]
*Babesia venatorum*	CGATTTCGCTTTTGGGATT	[12]
*Babesia vogeli*	AGCGTGTTCGAGTTTGCC	[41]
*Babesia vulpes* (1)	CCGAACGTAATTTTATTGATTTG	[12]
*Babesia vulpes* (2)	CTTATCATTAATTTCGCTTCCGAACG	[36]
*Borrelia burgdorferi* (*s.l*.)	CTTCCATCTCTAYTTTGCCAAT	[42]
*Borrelia burgdorferi* (*s.s*.)	AACACCAATATTTAAAAAACATAA	[19]
*Borrelia afzelii*	AACATTTAAAAAATAAATTCAAGG	[19]
*Borrelia bissetti*/*Borrelia carolinensis*	CACTAACATTTAAAAAATATAAAATAAAAT	[42]
*Borrelia garinii* (1)	AAAATCAATGTTTAAAGTATAAAAT	[43]
*Borrelia garinii* (2)	AACATGAACATCTAAAAACATAAA	This study
*Borrelia lusitaniae*	TTTTTAAATCAAACATTCAAAAAAAT	[42]
*Borrelia miyamotoi*	AGCACAACAGGAGGGAGTTCAAGC	[44]
*Borrelia spielmanii*	GTCAATATCTATTTTCTTTTTTATG	[42]
*Borrelia valaisiana* (1)	CATTAAAAAAATATAAAAAATAAATTTAAGG	[19]
*Borrelia valaisiana* (2)	CATGTCAATATCTATTTTATTTTTTACATTA	[42]
*Rickettsia* catch-all	TTTAGAAATAAAAGCTAATACCG	[45]
*Rickettsia conorii*	CTTGCTCCAGTTAGTTAGT	[45]
*Rickettsia helvetica*	GCTAATACCATATATTCTCTATG	[45]
*Rickettsia massiliae*	TGGGGCTTGCTCTAATTAGT	[46]
*Rickettsia raoultii*	CTAATACCGCATATTCTCTACG	[12]
“*Ca.* Midichloria mitochondria”	GCGAAATAACAGTTGGAAGCAAT	[18]

Oligonucleotide probes containing an N-terminal N-(trifluoracetamidohexyl-cyanoethyl,N,N-diisopropyl phosphoramidite [TFA])-C6 amino linker were synthesized by Thermo Fisher Scientific. Specific probes targeted 10 *Babesia* species. Furthermore, two catch-all *Theileria/Babesia* probes were included to capture possible unknown species or variants of species. In addition to one catch-all probe for *Ehrlichia/Anaplasma*, specific probes for *E. canis*, *Ehrlichia ewingii*, *A. phagocytophilum*, *Anaplasma platys*, and “*Candidatus* Neoehrlichia mikurensis” were also included.

For *Borrelia* species detection, *B. burgdorferi* (*s.l.*) was included as a catch-all probe together with specific probes for differentiating eight *Borrelia* species. Finally, *Rickettsia conorii*, *R. helvetica*, *R. massiliae*, *R. raoultii*, a catch-all probe for *Rickettsia* detection [22] plus a specific probe for “*Candidatus* Midichloria mitochondria” detection completed the membrane.

RLB hybridization was conducted as described previously [20]. In brief, a Biodyne C membrane was activated using 16% (wt/wv) 1-ethyl-3-(3-dimethyl-amino-propyl) carbodiimide (EDAC) (Carl Roth GmbH, Karlsruhe, Germany) for 10 min, after which the oligonucleotide probes were covalently linked to the membrane in 0.5 M NaHCO_3_ in a mini-blotter. Thereafter, the membrane was inactivated in 100 mM NaOH after washing in 2× SSPE/0.1% SDS at 60 °C and then stored in 20 mM EDTA, pH 8.0. For RBL assays, 10 µl of PCR product was added to 150 µl of 2× SSPE/0.1% SDS after denaturing at 100 °C for 10 min, followed by immediate cooling on ice. Denatured PCR products were subsequently hybridized to a Biodyne C membrane at 42 °C for 60 min. Thereafter, each membrane was washed twice in 2× SSPE/0.5% SDS at 50 °C for 10 min, incubated for 30 min at 42 °C in 2× SSPE/0.5% SDS with 5 µl of streptavidin-POD conjugate (Roche Diagnostic, Germany), again washed twice in 2× SSPE/0.5% SDS at 42 °C for 10 min, and finally washed twice in 2× SSPE for 5 min at room temperature. Hybridization detection was carried out by using chemiluminescence using Amersham ECL detection reagents [16].

## Results

### Tick collections

**Fig. 1:**
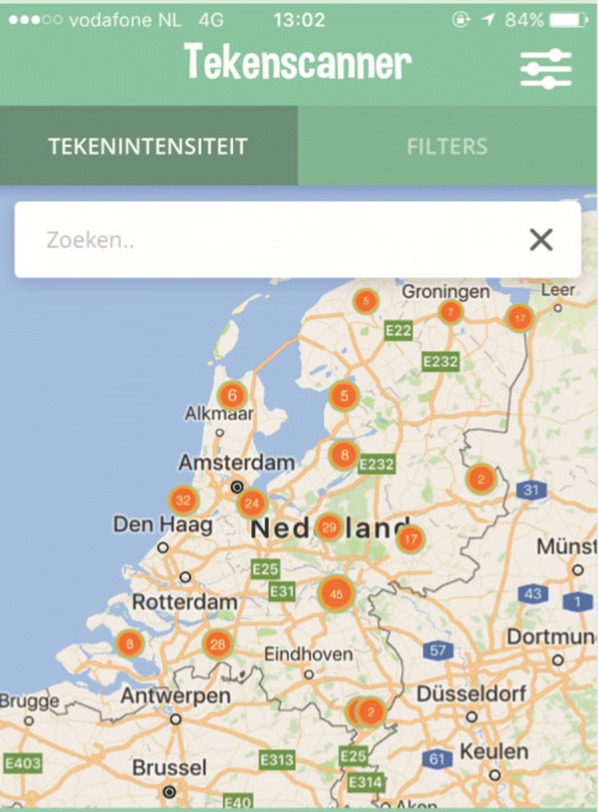
Screenshots of the “Tekenscanner” app showing some of the locations where ticks were found in the Netherlands

**Fig. 2:**
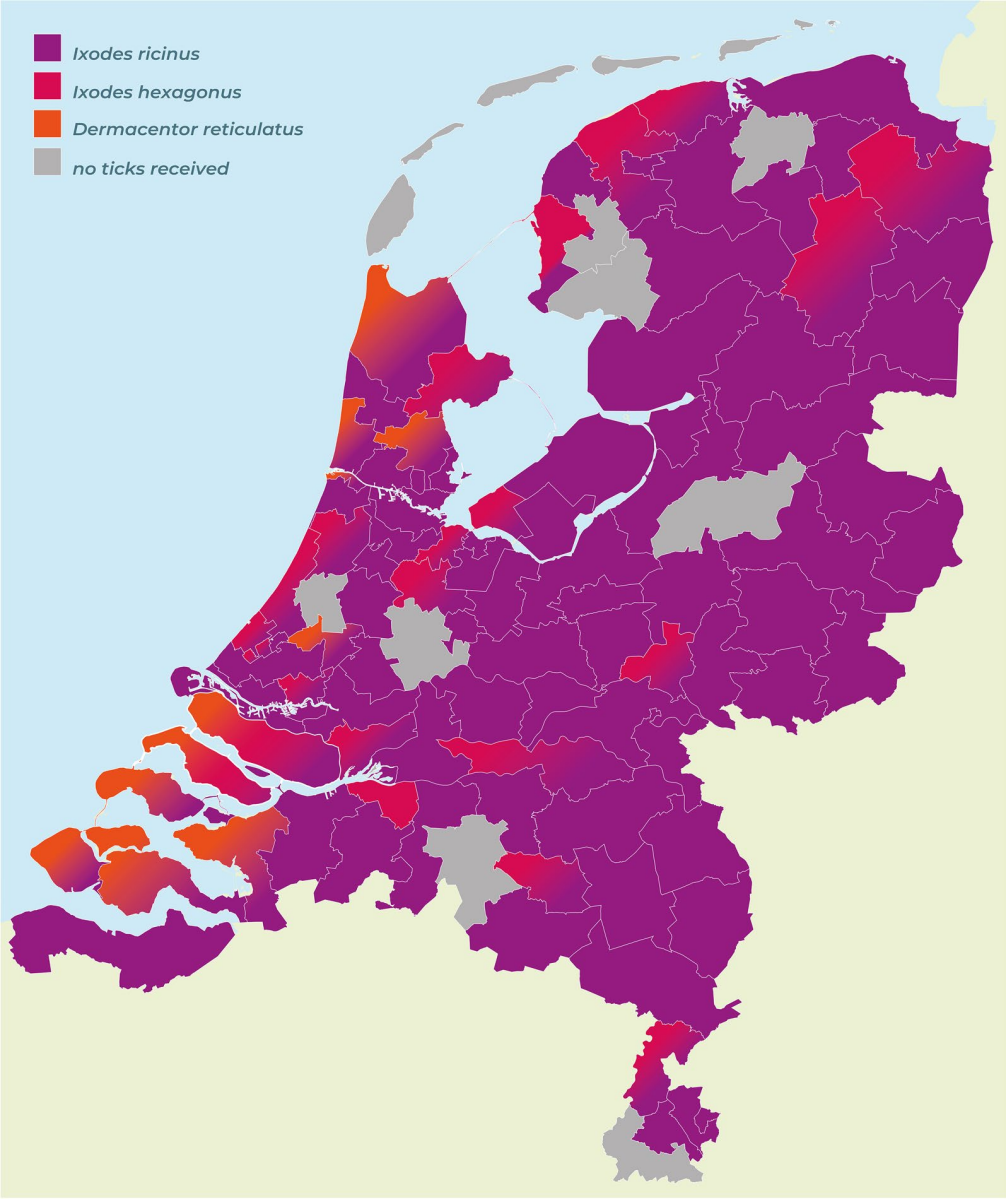
A distribution map of tick species recorded on dogs and cats based on postal codes of the Netherlands

The app was downloaded 5591 times and resulted in the collection of 1273 georeferenced and barcoded ticks, with a peak submission in May and June 2018. A screenshot of the app is provided as an illustration in Fig. 1. There were 1004 ticks removed from 406 dogs and 268 ticks removed from 111 cats. *Ixodes ricinus* was the predominant species (90%), with all stages found on dogs as well as on cats. *Ixodes hexagonus* (7.3%) female and nymphal ticks were also identified on both hosts, whereas adults of *D. reticulatus* (2.4%) and *R. sanguineus* (0.2%) were exclusively found on dogs. *Rhipicephalus sanguineus* ticks were removed from dogs that had travelled in France and the USA. A distribution map of tick species recorded on dogs and cats based on postal codes of the Netherlands is presented in Fig. 2.

### Pathogen detection

Almost 15% of the ticks recovered from dogs carried one or more pathogens, whereas 13.8% of the ticks removed from cats were infected. *Ixodes ricinus* collected from dogs contained *Borrelia* spp. (1.9%), *Babesia* spp. (0.7%), *A. phagocytophilum* (1.3%), “*Ca.* Neoehrlichia mikurensis” (2.9%) and *R. helvetica* (7.3%) (Table 2). *Ixodes ricinus* recovered from cats were infected with *Borrelia* spp. (1.9%), *Babesia* spp. (0.4%), *A. phagocytophilum* (1.9%), “*Ca.* Neoehrlichia mikurensis” (2.6%) and *R. helvetica* (6.7%). *Ixodes hexagonus* ticks (*n* = 93) collected primarily from cats, but also from dogs, all tested negative. *Dermacentor reticulatus* ticks were infected with *R. raoultii* (16%) and with *A. phagocytophilum* and were detected only in autumn (Table 3). *Rhipicephalus sanguineus* ticks were not infected (*n* = 4). *Rickettsia helvetica* was found in *I. ricinus* females together with *B. venatorum*, “*Ca.* Neoehrlichia mikurensis” or *Borrelia* species. Finally, seven *I. ricinus* ticks were recorded through the app by owners while travelling outside the Netherlands, e.g. in Belgium (4 ticks), Germany (1 tick), Italy (1 tick) and the Ukraine (1 tick). One tick from Italy was infected with “*Ca.* Neoehrlichia mikurensis”.

**Table 2:** Tick-borne pathogens detected in *Ixodes ricinus* and *Dermacentor reticulatus* ticks from dogs

	No. of positive *I. ricinus* (%)			No. of positive *D. reticulatus* (%)	
	Nymph (*n* = 25)	Male (*n* = 161)	Female (*n* = 696)	Male (*n* = 6)	Female (*n* = 25)
*Anaplasma phagocytophilum*	–	3 (0.3)	9 (1.0)	–	1 (3.2)
“*Ca.* Neoehrlichia mikurensis”	1 (0.1)	8 (0.9)	21 (2.4)	–	–
*Babesia microti*	–	–	3 (0.3)	–	–
*Babesia venatorum*	–	1 (0.1)	4 (0.4)	–	–
*Borrelia burgdorferi* (*s.s.*)	–	4 (0.4)	8 (0.8)	–	–
*Borrelia spielmanii*	–	2 (0.2)	2 (0.2)	–	–
*Borrelia garinii*	–	–	1 (0.1)	–	–
*Borrelia afzelii*	–	–	1 (0.1)	–	–
*Borrelia bisetti*	–	–	2 (0.2)	–	–
*Rickettsia helvetica*	1 (0.1)	11 (1.2)	65 (7.3)	–	–
*Rickettsia raoultii*	–	–	–	–	4 (12.9)

**Table 3:** Tick-borne pathogens detected in *Ixodes ricinus* from cats

	No. of positive *I. ricinus* (%)			
	Larva (*n* = 16)	Nymph (*n* = 14)	Male (*n* = 17)	Female (*n* = 214)
*Anaplasma phagocytophilum*	–	–	–	5 (1.9)
“*Ca.* Neoehrlichia mikurensis”	1 (0.4)	2 (0.8)	1 (0.4)	4 (1.5)
*Babesia venatorum*	–	–	1 (0.4)	–
*Borrelia spielmanii*	–	–	–	1 (0.4)
*Borrelia garinii*	–	–	2 (0.8)	–
*Borrelia valaisiana*	–	–	–	1 (0.4)
*Borrelia miyamotoi*	–	–	–	1 (0.4)
*Rickettsia helvetica*	–	–	1 (0.8)	18 (6.9)

## Discussion

Since the launch of the Tekenscanner app in April 2018, there were over 5000 downloads, which resulted in the collection of 1273 ticks. There were 1004 ticks removed from 406 dogs and 268 ticks from 111 cats. The lower number of ticks from cats *versus* dogs probably reflects differences in their behaviour and biology; however, there were no significant differences between the mean number of ticks collected from dogs *versus* cats (2.48 *vs* 2.41; *P* < 0.05).

Mapping predominant *I. ricinus* (90%) ticks together with *I. hexagonus* (7.3%) and *D. reticulatus* (2.4%) on a chart of the Netherlands divided into postal codes revealed specific clustering for *D. reticulatus*, whereas *Ixodes* ticks were much more widely distributed. However, this map is preliminary and requires improvement from more ticks collected during the forthcoming tick seasons (Fig. 2).

Importantly, *D. reticulatus* continues to broaden its distribution with novel locations since those already reported in 2015 [11] and 2016 [23]. New locations where dogs encountered these ticks remain to be surveyed to confirm the presence of significant populations of resident ticks in the vegetation. Although all *D. reticulatus* (*n* = 31) collected from dogs submitted thus far were negative for *B. canis*, the infection may be present in field ticks. Previously, ticks collected from novel foci were indeed found infected with *B. canis*, whereas all ticks removed from dogs visiting those foci tested negative [11].

It was found that nearly 15% of the ticks from dogs carried one or more pathogens, whereas 13.8% of the ticks from cats were infected. Almost 2% of *I. ricinus* collected from dogs (Table 2) and from cats (Table 3) contained six different *Borrelia* species. A similar diversity of *Borrelia* species was found in a previous study conducted in the Netherlands more than a decade ago, although the percentage of ticks (7.2%) harbouring spirochetes belonging to the *B. burgdorferi* (*s.l*.) group was higher [12]. Furthermore, *B. microti* and *B. venatorum*, two parasites with possible zoonotic implications, were detected in ticks derived from dogs and cats at a similar frequency as reported previously [12]. Finally, between 1–2% of ticks carried *A. phagocytophilum* in both studies (Tables 2, 3) [12].

In this study, *I. ricinus* (*n* = 1145) were tested in a pooled sample structure containing an average of 4.5 ticks, whereas in other studies ticks (*n* = 251) were previously tested individually [12]. Although the methods used in both studies differ, the results are quite similar. However, it is possible that pooling of ticks has masked additional infections that would have been detected if the ticks were tested individually. Since there is no standardized procedure, both approaches are justified, but direct comparison is limited. There are many other approaches used in the literature. For instance, Claerebout et al. [6] selected one tick (nymphs or adult) for DNA analysis, but when different tick species were present on the same host, one tick of each tick species was randomly selected for analysis. Another approach was followed by Geurden et al. [9] who pooled all ticks between one and 10 ticks of the same species.

It is interesting to note that despite the continuous challenge of companion animals by infected ticks, clinical cases of borreliosis, anaplasmosis and babesiosis are relatively rare. A thorough discussion of Lyme borreliosis in dogs and cats is beyond the scope of this paper. However, it is worth mentioning that there is much to gain by applying available serological and molecular tests combined with clinical observations and known infectious tick challenges as conducted in the UK [13] and elsewhere in Europe [24].

As far as canine anaplasmosis in the Netherlands is concerned, a recent study clearly demonstrated subclinical and clinical *A. phagocytophilum* infections in a pack of resident Rhodesian ridgeback dogs [25]. At least one additional clinical case with typical cytoplasmic inclusion bodies in circulating neutrophils was confirmed in a dog diagnosed in a veterinary clinic in The Hague in the Netherlands (F. Jongejan, unpublished data, 2015).

As far as Spotted Fever Group rickettsiae are concerned, *R. helvetica* was co-infecting *I. ricinus* female ticks together with *B. venatorum*, “*Ca.* Neoehrlichia mikurensis” or *Borrelia* species. Sixteen percent of *D. reticulatus* ticks collected from dogs in this study were infected with *R. raoultii* (Table 3), which is similar to 14% of those ticks reported positive in 2007 [12]. Likewise, “*Ca.* Neoehrlichia mikurensis” [26] has been identified in approximately 2–3% of all *I. ricinus* ticks in this study, confirming a similar percentage documented a decade ago [12].

Our current range of probes do encompass all *Anaplasma*, *Ehrlichia* and *Borrelia* species as well as all *Babesia* and *Theileria* species, and if DNA is amplified which does not hybridize with one of the species-specific probes, sequencing of the catch-all signal will determine whether there is a variant of an existing species or even a new species involved. This is key to RLB, which has resulted in the discovery of *Babesia bicornis* and *Theileria bicornis* [16]. Interestingly, clinical cases of *Cytauxzoon* have recently been reported in cats in several western European countries [27]. Moreover, *Hepatozoon canis* associated with the ingestion of ticks by dogs has very recently been reported from the UK [28]. New probes designed to facilitate parasite detection using RLB (Table 1) are currently expanded to include probes for the detection and differentiation of *Cytauxzoon* and *Hepatozoon* species. Screening of extracted DNA from ticks targeting those additional species is ongoing.

The role of companion animals in the dissemination of ticks and consequently possible tick-borne pathogens needs to be taken into further consideration. Seven *I. ricinus* ticks were recorded through the app by owners while travelling outside the Netherlands. This highlights the international travel of tick species with their hosts within Europe. Moreover, one of the *R. sanguineus* ticks that was reported through the app had entered the Netherlands on a dog from Texas, USA. In a comprehensive review, Fooks & Johnson [29] discussed the zoonotic risks of the international travel of pets and correctly mentions both *R. sanguineus* as well as *D. reticulatus* ticks which could possibly accompany these jet-set pets [29]. However, the possibility that the Asian longhorned tick, *Haemaphysalis longicornis*, could also have travelled on dogs from Asia and then been introduced into the USA was never contemplated. Now, this tick has already invaded nine different states in the USA [30, 31].

It is relevant to discuss here the possible scenarios with respect to the outbreaks of canine babesiosis, caused by *B. canis*, in southern England [32]. The probability that an asymptomatic dog entering into the UK, which subsequently infects a local population of *D. reticulatus* ticks is lower than that of a *Babesia canis*-infected *Dermacentor reticulatus* female tick being introduced by a dog. If the infected engorged tick drops into fertile soil, adults of the subsequent generation will readily transmit the potentially fatal infection to passing dogs. This is what most likely also happened in the outbreak of babesiosis in the Netherlands.

In any case, identification of ticks on companion animals is of prime importance. If this is done through the companion animal owner app, a link between a positive (introduced) tick and a potential patient can be established quickly. On the other hand, in most traditional surveys, this link is completely lost since usually ticks are tested years after they are collected.

Another example of the use of a smartphone app was recently evaluated for the prevention of tick bites in the Netherlands [33] and subsequently further analysed [34, 35]. It was concluded that this app facilitated an increase in public awareness, although the actual ticks were not identified and a link between people bitten by ticks and laboratories testing them was not established.

Importantly, in studies wherein tick surveillance depends on community engagement, there is a bias towards individuals who decide to participate versus those that discard the tick in disgust. Further public awareness on the usefulness of the approach through social media and rapid feedback of results are factors expected to increase the number of ticks reported in the upcoming tick seasons. Finally, the positive experience with the Tekenscanner app in the Netherlands has created opportunities to continue and launch the app as part of a coordinated European tick and tick-borne pathogen surveillance programme. This will include an early warning system for exotic ticks with the ultimate aim to improve control of ticks and associated diseases in companion animals.

## Conclusions

The launch of the tekenscanner app stimulated companion animal owners to operate our tick and tick-borne pathogen surveillance programme. Feedback of the results into the app was formatted as a map of ticks in the Netherlands. The Dutch tick fauna is dominated by *I. ricinus*, which is prevalent throughout the country, whereas *I. hexagonus* is more restricted. *Dermacentor reticulatus* is continuing its spread into novel areas, which justifies year-round tick control measures, in particular, because adult *D. reticulatus* are active outside the regular tick season dominated by *I. ricinus* ticks. Our preliminary findings concur with those published a decade ago and confirm that a broad spectrum of tick-borne pathogens is established in the Netherlands, including several zoonotic pathogens.

## Abbreviations

RLB: reverse line blot
SDS: sodiumdodecylsulfate
PCR: polymerase chain reaction
Streptavidine-POD: streptavidine-peroxidase
SSPE: sodium chloride-sodium phosphate-EDTA
